# Antileishmanial Activities of Medicinal Herbs and Phytochemicals In Vitro and In Vivo: An Update for the Years 2015 to 2021

**DOI:** 10.3390/molecules27217579

**Published:** 2022-11-04

**Authors:** Abdalla A. Hassan, Hassan E. Khalid, Abdelwahab H. Abdalla, Maowia M. Mukhtar, Wadah J. Osman, Thomas Efferth

**Affiliations:** 1 Faculty of Pharmacy, University of Khartoum, Khartoum 11115, Sudan; 2 Faculty of Agriculture, University of Khartoum, Khartoum 11115, Sudan; 3 Tropical Medicine Institute, University of Khartoum, Khartoum 11115, Sudan; 4 Department of Pharmacognosy, Faculty of Pharmacy, Prince Sattam bin Abdulaziz University, Alkharj 11942, Saudi Arabia; 5 Department of Pharmaceutical Biology, Institute of Pharmaceutical and Biomedical Sciences, Johannes Gutenberg University, Staudinger Weg 5, 55128 Mainz, Germany

**Keywords:** *Leishmania*, medicinal plant, natural product, neglected tropical disease, phytotherapy, pharmacognosy, promastigotes

## Abstract

Leishmaniasis is one of the most neglected tropical diseases that present areal public health problems worldwide. Chemotherapy has several limitations such as toxic side effects, high costs, frequent relapses, the development of resistance, and the requirement for long-term treatment. Effective vaccines or drugs to prevent or cure the disease are not available yet. Therefore, it is important to dissect antileishmanial molecules that present selective efficacy and tolerable safety. Several studies revealed the antileishmanial activity of medicinal plants. Several organic extracts/essential oils and isolated natural compounds have been tested for their antileishmanial activities. Therefore, the aim of this review is to update and summarize the investigations that have been undertaken on the antileishmanial activity of medicinal plants and natural compounds derived, rom plants from January 2015 to December 2021. In this review, 94 plant species distributed in 39 families have been identified with antileishmanial activities. The leaves were the most commonly used plant part (49.5%) followed by stem bark, root, and whole plant (21.9%, 6.6%, and 5.4%, respectively). Other plant parts contributed less (<5%). The activity was reported against amastigotes and/or promastigotes of different species (*L. infantum*, *L. tropica, L. major*, *L. amazonensis*, *L. aethiopica*, *L. donovani*, *L. braziliensis*, *L. panamensis*, *L. guyanensis*, and *L. mexicana*). Most studies (84.2%) were carried out in vitro, and the others (15.8%) were performed in vivo. The IC_50_ values of 103 plant extracts determined in vitro were in a range of 0.88 µg/mL (polar fraction of dichloromethane extract of *Boswellia serrata*) to 98 µg/mL (petroleum ether extract of *Murraya koenigii*). Among the 15 plant extracts studied in vivo, the hydroalcoholic leaf extract of *Solanum havanense* reduced parasites by 93.6% in cutaneous leishmaniasis. Voacamine extracted from *Tabernaemontana divaricata* reduced hepatic parasitism by ≈30 times and splenic parasitism by ≈15 times in visceral leishmaniasis. Regarding cytotoxicity, 32.4% of the tested plant extracts against various *Leishmania* species have a selectivity index higher than 10. For isolated compounds, 49 natural compounds have been reported with anti-*Leishmania* activities against amastigotes and/or promastigotes of different species (*L. infantum*, *L. major, L. amazonensis*, *L. donovani* and *L. braziliensis).* The IC_50_ values were in a range of 0.2 µg/mL (colchicoside against promastigotes of *L. major*) to 42.4 µg/mL (dehydrodieuginol against promastigotes of *L. amazonensis*). In conclusion, there are numerous medicinal plants and natural compounds with strong effects (IC_50_ < 100 µg/mL) against different *Leishmania* species under in vitro and in vivo conditions with good selectivity indices (SI > 10). These plants and compounds may be promising sources for the development of new drugs against leishmaniasis and should be investigated in randomized clinical trials.

## 1. Introduction

Leishmaniasis is a group of diseases caused by protozoa parasites from more than 20 Leishmania species. In 2018, 92 countries and 83 territories were considered endemic for Leishmania species or had previously reported cases of cutaneous and visceral leishmania, respectively. Today, more than 1 billion people live in areas endemic to leishmaniasis and are at risk of infection. An estimated 30,000 new cases of visceral leishmania and more than 1 million new cases of cutaneous leishmania occur annually [1]. The parasite is categorized into two main groups: Old World leishmaniasis, which is endemic in Africa, Asia, the Mediterranean, and the Middle East. *Leishmania tropica*, *L. major*, *L. aethiopica*, and *L. donovani* are the four common species causing Old World leishmaniasis. New World leishmaniasis is caused by *L. mexicana*, *L. amazonensis*, *L. braziliensis*, *L. panamensis*, *L. peruviana*, *L. guyanensis*, *L. pifanoi*, *L. venezuelensis*, *L. shawi*, and *L. lainsoni* [2]. There are three clinical forms of leishmaniasis in humans: namely, cutaneous, mucocutaneous and visceral leishmaniasis. Cutaneous leishmaniasis is a less severe form of the disease which usually manifests in self-healing ulcers. Mucocutaneous leishmaniasis results in disfiguring lesions of mucous membranes in the nose, mouth, and throat. Visceral leishmaniasis is the most severe form of the disease which can result in 95% mortality of infected patients if not treated [3].

In 2020, more than 90% of new cases of visceral leishmaniasis reported to the WHO occurred in Bangladesh, Brazil, China, Ethiopia, Eritrea, India, Kenya, Somalia, South Sudan, Sudan, and Yemen [1]. Over 90% of mucocutaneous leishmaniasis occurred in Bolivia, Brazil, Ethiopia, and Peru, and more than 85% of cutaneous leishmaniasis cases appeared in Afghanistan, Algeria, Brazil, Colombia, Iran, Libya, Pakistan, Peru, Syria, and Tunisia [1]. Depending on the stage of its life cycle, the parasite exhibits two morphological forms in its life cycle: The amastigotes in macrophages of the mammalian host and the promastigotes in the gut of the sand fly vectors. The life cycle of the Leishmania parasite starts if a parasitized female sand fly takes a blood meal from a vertebrate host to produce its eggs. As the sand fly feeds, infective promastigotes enter the vertebrate host via the insect’s proboscis. The promastigotes are then phagocytosed by macrophages which they transform into amastigotes and reproduce by binary fission. They increase in number until the cell eventually bursts and then infects other phagocytic cells to continue the cycle [4]. Over the years, a number of drugs have been employed for the treatment of leishmaniasis. A brief account of the mechanism of action and mode of administration of these drugs has been presented in Table 1 [5].

Latest developments in the prevention and treatment regarding a permanent solution for leishmaniasis in terms of successful human vaccination is still a major challenge. However, there are different vaccinations currently being tested in mouse models. One of them uses “killed but metabolically active” parasites to induce host immune system reaction. Using salivary peptides of the sandfly holds the potential to be used as a vaccine component. However, the complex immune response makes it a challenge [6]. Macrophage-targeted drug delivery systems are another novel approach to directly affect *Leishmania* parasites that live in the macrophages. As getting into macrophages is a challenge, liposomes, microspheres, nanoparticles, and carbon nanotubes are some of the various drug carriers that are studied to target macrophages. In addition, the use of specific receptors expressed by macrophages to actively deliver a drug is also used [7].

The current treatment by chemical drugs has several limitations such as toxic side effects, high costs, frequent relapses, the development of resistance, and the requirement for long-term treatment [8,9]. Thus, investments in novel drug development against this parasitic disease may be a risky affair. Medicinal plants are centuries-old sources in the various traditional herbal medicine systems of the world. For instance, their importance lies in the fact that the WHO concludes that about 80% of the world’s population relies on them for primary health care [10]. Moreover, 25 to 50% of the pharmacopeias worldwide contain plant products and drugs derived from natural products [11]. Therefore, current research approaches for the treatment of leishmaniasis should largely consider medicinal plants as an important area of search.

The aim of this review is to update and summarize the investigations that have been undertaken on the antileishmanial activity of medicinal plants and natural compounds derived from plants from January 2015 to December 2021.

## 2. Results

As shown in Table 2, 92 plant species distributed in 39 families have been identified with anti-*Leishmania* activities. The family *Fabaceae* accounted for the highest percentage (9.7%) followed by *Asteraceae* (7.6%). *Lamiaceae* and *Solanaceae* account for 6.5% each.

**Figure 1:**
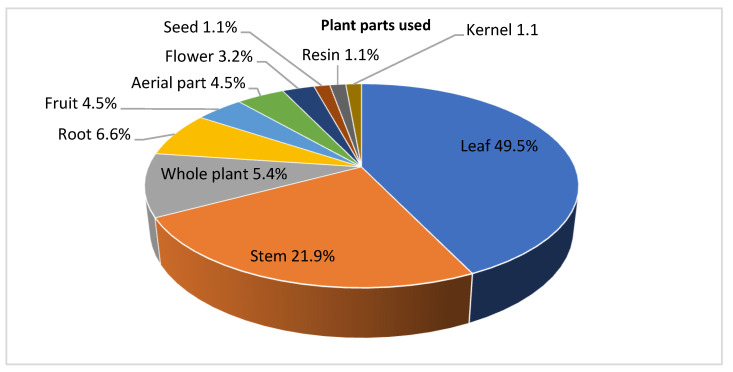
Fraction of plant parts used in anti-*Leishmania* studies.

The leaves were the most commonly used plant part as compared to other parts (49.5%) followed by stem bark, roots, and whole plant (21.9%, 6.6%, and 5.4%, respectively). Aerial parts and fruits accounted for 4.5% each. Other plant parts (flowers, seeds, resins, branches, and kernels) contributed less (<4%) (Figure 1).

With respect to the test methods, 84.2% of studies were carried in vitro, while 15.8% of them were performed using in vivo assays (Table 3 and Table 4). For in vitro assay, 80 medicinal plants were screened in vitro for antileishmanial activities against different *Leishmania* species (*L. infantum*, *L. tropica*, *L. major*, *L. amazonensis*, *L. aethiopica*, *L. donovani*, *L. braziliensis*, *L. panamensis*, *L. guyanensis*, *and L. mexicana*) and life cycle forms (amastigotes and/or promastigotes). The IC_50_ value of 103 plant extracts/essential oils determined in vitro was in a range of 0.88 µg/mL (polar fraction of dichloromethane extract of *Boswellia serrata*) to 98 µg/mL (petroleum ether extract of *Murraya koenigii*) (Table 3). *B. serrata* (resins), R*. officinalis* (leaves), *A. riparia* (fruits), *M. pulegium* (leaves) as extracts had strong anti-*Leishmania* activity (0.88, 1.2, 1.3, and 1.3 µg/mL, respectively).

For in vivo assay, among the 15 medicinal plants studied in vivo, the highest activity against cutaneous leishmaniasis was exhibited by the hydroalcoholic leaf extract of *Solanum havanense*, which reduced parasites by 93.6%, and the highest activity against visceral leishmaniasis was shown by the voacamine compound extracted from *Tabernaemontana divaricata,* which reduced the hepatic and splenic parasitism by ≈30 times and ≈15 times, respectively (Table 4). For cytotoxic activity, 32.4% of tested plant extracts have good cytotoxic activity with a selectivity index of SI > 10. (Table 5).

For isolated compounds, 49 natural compounds have been identified with anti-*Leishmania* activities against amastigotes and/or promastigotes of different species (*L. infantum*, *L. major*, *L. amazonensis*, *L. donovani and L. braziliensis).* The IC_50_ values were in the range of 0.2 µg/mL (colchicoside against promastigotes of *L. major*) to 42.4 µg/mL (dehydrodieuginol against promastigotes of *L. amazonensis*) (Table 6).

Numerous natural compounds were isolated from different parts of the plants that were used in traditional medicine to treat leishmaniasis [67]. These compounds act against *Leishmania* by various mechanisms including the disintegration of cytoplasmic membranes, electron flow disturbances, active transport of crucial substances, coagulation of the cell contents, and destabilization of proton motive forces [68]. For example:

- Some medicinal plants are enriched with essential oils composed of different hydrophobic molecules which can diffuse easily across cell membranes and consequently gain access to intracellular targets [67,69]. They may also act on ATPases and other proteins located in cytoplasmic membranes that are surrounded by lipid molecules. They can also cause a distortion of lipid–protein interactions in hydrophobic parts of the proteins, or they can interact with the enzymes involved in the synthesis of structural sections.
- The diversity of terpenoids increases their biological activity spectrum, including several *Leishmania* species [70]. Terpenes can easily penetrate the lipid bilayer of the cell membrane and produce changes in the integrity of cell structure and the mitochondrial membrane of *Leishmania* parasites [67]. For example, Artemisinin induced apoptosis, depolarization of the mitochondrial membrane potential, and DNA fragmentation [71,72]. Ursolic acid induce programmed cell death independent of caspase 3/7 but dependent on mitochondria. The compound reduced the lesion size and parasite load of cutaneous leishmaniasis in vivo [70]. (−)-α-Bisabolol induced phosphatidylserine externalization and caused cytoplasmic membrane damage, both of which are apoptosis indicators. The compound also decreased ATP levels and disrupted the mitochondrial membrane potential [73].
- Plants enriched with antioxidant compounds such as flavonoids may act by initiating morphological changes and causing a loss of cellular integrity, leading to cell cycle arrest in the G1 phase [59]. They also may act by damaging the mitochondria of the parasites [67]. For example, apigenin increased intracellular reactive oxygen species (ROS) and the number of double-membrane vesicles as well as myelin-like membrane inclusions, which are characteristics of the autophagic pathway. Furthermore, the fusion between autophagosome-like structures and parasitophorous vacuoles was observed [65]. Epigallocatechin 3-*O*-gallate (EGCG) has increased ROS levels, which decreased the mitochondrial membrane potential and the ATP levels [58].
- The diversity of structures within the coumarin group enables them to exhibit many biological activities, including anti-*Leishmania* activity. It represents a promising natural compound that can act on two fronts: as a treatment for leishmaniasis (able to induce mitochondrial membrane damage and changes in ultrastructure [74] and as a tool to control *Leishmania* vectors (might block the transmission of leishmaniasis since they decrease parasite loads [27].
- Many alkaloids have been described as having biological activities against trypanosomatids, such as *Leishmania* spp. For example, heterocyclic steroids (solamargine and solasonine) induced different immunochemical pathways in macrophages and dendritic cells. Additionally, they were capable of enhancing the expression levels of transcription factors, such as NFκB/AP-1 [43]. In addition, isoquinoline alkaloid (berberine) has leishmanicidal activity through a reduction in the viability of promastigotes and the generation of ROS in these cells. It also increased the levels of mitochondrial superoxide and induced the depolarization of mitochondrial transmembrane potential [53].

## 3. Methods

### 3.1. Study Design and Setting

In order to perform this review, the following aspects were addressed: identification and selection of the theme of the research question, establishment of criteria for selection of the sampling, the definition of information to be extracted from selected studies, assessment of the studies included in the integrative review, and final explanation of the results.

### 3.2. Search Strategies

The databases used for this article were PubMed, Google Scholar, Web of Science, Research Gate, SCOPUS, and Scientific Electronic Library Online (SciELO) using the keywords: neglected tropical disease, *Leishmania* species, anti-*Leishmania* activity, natural product, medicinal plants, and promastigote form. We used the search terms separately and in combination with the Boolean operators “OR” or “AND”.

### 3.3. Inclusion and Exclusion Criteria

The initial total articles (1374) were adjusted for the restriction in the year of publication (from 1 January 2015 to 31 December 2021) (806), duplicates (273), articles that were not available in full (67) and articles in other languages (4). After a review of their titles and abstracts, some articles were discarded, since the anti-leishmanial activity (IC_50_) values were higher than 100 µg/mL (134), and they tested extract/natural compounds obtained through other natural sources (algae, fungi, etc.) (11). The full texts of the remaining articles were reviewed in detail. However, further articles were discarded after the full text had been reviewed (18) since they did not address much of the required information. Finally, 61 articles were evaluated as valuable to reach the goals of this review. The methodological validity of all 61 studies was proven prior to inclusion in the review by undertaking a critical appraisal using a standardized instrument [75].

### 3.4. Data Extraction and Analysis

The data extraction protocol included the scientific and family names, parts of the plant used, most active extract/ essential oil employed in the experiment, name of natural compound, *Leishmania* species and form, IC_50_ values, potential groups/compounds responsible for activity, clinical form of leishmaniasis, route, the dose of administration and scheme of treatment, the efficacy of the treatments in the experiment, cytotoxic activity, selectivity index, the authors, and year of publication. In the results analysis, an active extract/compound was considered if the IC50 value was less than or equal to 10 µg/mL against the promastigote or amastigote forms. Moderate activity was defined if the IC50 was greater than 10 and less than 50 µg/mL and weakly active if the IC50 value was greater than 50 µg/mL and less than 100 µg/mL.

## 4. Conclusions and Perspectives

Leishmaniasis threatens about 350 million people around the world and continues to represent a menace on a global scale. Without a doubt, it requires utmost attention due to the lack of vaccines for the prevention and reported resistance against available chemical drugs for treatment. The intolerably high incidence of millions of new cases of leishmaniasis per year worldwide and deficiencies in current treatment point to an urgent need for new medications.

As a means to facilitate the accessibility of information, this review updates and summarizes recent results on medicinal plants and natural compounds against different *Leishmania* species. The plants presented here have demonstrated a diverse range of activities against different forms of leishmaniasis with some showing high activities that could be reasonable starting points for the further development of effective and affordable novel drugs.

However, it was also evident that the majority of experiments were performed with the promastigote form. We believe that these studies are undoubtedly important because promastigotes are infectious to man and other animals. However, it is urgent that future studies should be conducted to find compounds with anti-amastigote activity too, since the morbimortality associated with *Leishmania* is caused by this form.

It Is pleasing that more and more investigations report on the anti-*Leishmania* activity in vivo and more studies are needed in this respect, increasing the number of potential candidate compounds for further drug development. In vitro studies are valuable for the screening of extracts and isolated compounds as well as for investigations of the cellular and molecular modes of action. Since many natural compounds are rapidly metabolized in the human body by liver enzymes and gastrointestinal microflora, animal experiments are indispensable to identify candidates with sufficient half-life times in vivo and anti-*Leishmania* activities in concentration ranges that are reachable in the human blood. However, in the literature inspected by us, only four plants and two natural compounds have been investigated both in vitro and in vivo, i.e., *Prosopis juliflora* [34], *Ziziphus spina-christi* [37], *Piper pseudoarboreum* [33], and *Croton caudatus* [76] as well as epigallocatechin 3-O-gallate [58] and apigenin [65], respectively. More investigations are required to allow a direct comparison of in vitro and in vivo data.

Further down this line of argumentation, standardized extracts and/or isolated phytochemicals need to be tested in randomized clinical trials. Without convincing clinical evidence on safety and efficacy, preparations from traditional medicine will hardly reach considerable recognition in the medical world.

## Figures and Tables

**Table 1:** Drugs used for the treatment of leishmaniasis.

Name of the Drug	Mode of Action	Mode of Administration	Adverse Effects
Pentavalent antimonials	Inhibition of glycolysis and β-oxidation of fatty acids of parasite	Intralesional for CL, Parenteral	Abdominal pain, erythema, nausea, toxicity (hepatic, pancreas, renal, muscular, and skeletal cardiothrombocytopenia or leukopenia)
Amphotericin B	Binding to parasite’s membrane sterols and changing its permeability selective to K^+^ and Mg^2+^	Liposomal formulations, Deoxycholate formulations	Fever, nausea, hypokalemia, anorexia, leukopenia, kidney failure, and heart problems
Pentamidine	Interferes with DNA synthesis and modifies the morphology of kinetoplast	Parenteral, Intramuscular administration	Pain, nausea, vomiting, dizziness, myalgia, hypertension, headache, hypoglycemia, and transient hyperglycemia
Miltefosine	Associated with phospholipid biosynthesis and alkyl-lipid metabolism in leishmania	Oral for VL	Nausea, vomiting, diarrhea, and raised creatinine
Paromomycin	Inhibition of protein biosynthesis in sensitive organism	Topical for CL Parenteral for VL	Erythema, pain, edema, and ototoxicity (damage to the internal ear)

**Table 2:** Botanical characteristics of the medicinal herbs in the present study.

No	Family Name	Scientific Name	Part Used
**1.**	Anacardiaceae	*Pistacia lentiscus*	Leaves
		*Schinus terebinthifolia*	Fruits
		*Schinus molle*	Leaves
		*Spondias mombin*	Leaves
**2.**	Annonaceae	*Annona senegalensis*	Stem bark
		*Bocageopsis multiflora*	Leaves
		*Guatteria latifolia*	Branch
		*Cleistopholis patens*	Stem bark
**3.**	Apiaceae	*Ferula communis*	Whole plant
**4.**	Apocynaceae	*Tabernaemontana divaricata*	Voacamine
		*Mondia whitei*	Roots
		*Pentalinon andrieuxii*	Pentalinon sterol
**5.**	Araliaceae	*Oreopanax floribundus*	Leaves
**6.**	Arecaceae	*Phoenix dactylifera*	Kernel and date fruit
**7.**	Asteraceae	*Acanthospermum hispidum*	Whole plant
		*Tessaria integrifolia*	Leaves
		*Abuta grandifolia*	Leaves
		*Cynara scolymus*	Leaves
		*Artemisia absinthium*	Leaves
		*Artemisia campestris*	Leaves
		*Artemisia herba-alba*	Aerial parts, Leaves
		*Bidens pilosa*	Whole plant
		*Tessaria integrifolia*	Whole plant
**8.**	Balanophoracea	*Thonningia sanguinea*	Whole plant
**9.**	Bignoniaceae	*Handroanthus serratifolius*	Lapachol
		*Jacaranda glabra*	Bark
**10.**	Burseraceae	*Boswellia serrata*	Resin
**11.**	Cannabaceae	*Celtis australis*	Leaves
**12.**	Capparaceae	*Capparis spinosa*	Fruits
**13.**	Cistaceae	*Citrus sinensis*	Leaves
**14.**	Combretaceae	*Terminalia ivorensis*	Leaves
**15.**	Cupressaceae	*Juniperus excelsa*	Leaves, fruits
**16.**	Ericaceae	*Arbutus unedo*	Leaves
		*Erica arborea*	Flower
**17.**	Euphorbiaceae	*Bridelia ferruginea*	Leaves
		*Ejije bidu*	Leaves
		*Croton caudatus*	Leaves
**18.**	Fabaceae	*Afzelia africana*	Stem bark
		*Baphia nitida*	Stem bark
		*Cassia alata*	Leaves
		*Cassia gloca*	Leaves
		*Cassia sieberiana*	Roots, leaves
		*Prosopis laevigata*	Leaves
		*Parkia clappertoniana*	Stem bark, leaves
		*Tamarindus indica*	Leaves
		*Prosopis juliflora*	Leaves
**19.**	Gentianaceae	*Anthocleista nobilis*	Leaves, stem bark, root
		*Centaurium erythraea*	Flowering, stems
**20.**	Lamiaceae	*Marrubium vulgare*	Leaves
		*Mentha pulegium*	Leaves
		*Otostegia integrifolia*	Whole plant
		*Rosmarinus officinalis*	Leaves
		*Salvia clandestina*	Aerial parts
		*Vitex fosteri*	Stem bark, leaves
**21.**	Lauraceae	*Aniba riparia*	Fruits
		*Persea ferruginea*	Leaves
		*Cinnamomum cassia*	Bark
**22.**	Loranthaceae	*Loranthus europaeus*	Aerial part
**23.**	Malvaceae	*Ceiba pentandra*	Stem bark
		*Cola acuminata*	Stem bark
		*Cola cordifolia*	Stem bark, leaves
		*Glyphaea brevis*	Leaves
**24.**	Marantaceae	*Thalia geniculata*	Roots
		*Iresine diffusa*	Flower
**25.**	Meliaceae	*Khaya grandifoliola*	Stem bark
		*Cedrela* spp	Bark
		*Azadirachta indica*	Leaves
**26.**	Moraceae	*Treculia africana*	Stem bark
		*Ficus capensis*	Stem bark, leaves
**27.**	Myrtaceae	*Eugenia uniflora*	Leaves, seed
**28.**	Ochnaceae	*Lophira lanceolata*	Stem bark, roots
**29.**	Olacaceae	*Ximenia americana*	Stem and twigs
**30.**	Papaveraceae	*Argemone mexicana*	Aerial parts
**31.**	Piperaceae	*Piper pseudoarboreum*	Leaves
**32.**	Rhamnaceae	*Ziziphus spina-christi*	Whole plant
**33.**	Rosaceae	*Pyrus communis*	Leaves
		*Pyrus pashia*	Leaves
		*Prunus armeniaca*	Leaves
		*Eryobotrya japonica*	Leaves
**34.**	Rubiaceae	*Mitragyna inermis*	Stem bark, leaves
		*Psychotria buhitenii*	Leaves
**35.**	Rutaceae	*Zanthoxylum zanthoxyloides*	Roots, stem bark
		*Murraya koenigii*	Stem bark
		*Clausena anisata*	Roots
**36.**	Scrophulariaceae	*Scoparia dulcis*	Aerial part
		*Licania salicifolia*	Leaves
**37.**	Solanaceae	*Solanum havanense*	Leaves
		*Solanum lycocarpum*	Leaves
		*Solanum myriacanthum*	Leaves
		*Solanum nudum*	Leaves
		*Physalis angulata*	Flowers
		*Solanum seaforthianum*	Leaves
**38.**	Urticaceae	*Urtica dioica*	Leaves
**39.**	Verbenaceae	*Lantana camara*	Leaves

**Table 3:** Anti-*Leishmania* activity of medicinal plants in vitro.

No.	Scientific Name	Organism	Stage	Part Used	Most Active Extract/ Essential Oil	IC50 (µg/mL)	Bioactive Compounds	Data Analysis (Activity)	Reference
**1.**	*Abuta * *grandifolia*	*L. amazonensis*	Promastigotes	Leaves	Ethanol	38.1	Alkaloids, triterpenes, saponins	Moderate	[12]
		*L. braziliensis*				31.1		Moderate	
**2.**	*Acanthospermum hispidum*	*L. donovani*	Promastigotes	Whole plant	50% aqueous ethanol	32.10	Essential oil, alkaloids	Moderate	[13]
**3.**	*Afzelia africana*	*L. donovani*	Promastigotes	Stem bark	50% aqueous ethanol	77.10	Alkaloids, tannins, flavonoids, saponins	Weak	[13]
**4.**	*Aniba riparia*	*L. amazonensis*	Amastigotes	Fruits	50% aqueous ethanol	1.30	Riparin E	High	[14]
			Promastigotes			4.70		High	
**5.**	*Annona * *senegalensis*	*L. donovani*	Promastigotes	Leaves	50% aqueous ethanol	10.80	Alkaloids, tannins, flavonoids, saponins, terpenoids, glycosides	Moderate	[13]
				Stem bark		27.80		Moderate	
**6.**	*Anthocleista * *nobilis*	*L. donovani*	Promastigotes	Leaves	50% aqueous ethanol	41.50	Glycosides, saponins, steroids	Moderate	[13]
				Root		79.0	Anthocleistol	Weak	
**7.**	*Arbutus unedo*	*L. infantum*	Promastigotes	Leaves	n-Hexane	64.05	Phenolics, flavonoids	Weak	[15]
		*L. tropica*				79.57		Weak	
**8.**	*Argemone * *mexicana*	*L. donovani*	Promastigotes	Aerial part	Petroleum ether	50.0	-	Moderate	[16]
**9.**	*Artemisia * *absinthium*	*L. major*	Promastigotes	Leaves	Hydrodistillation	1.49	Essential oil	High	[17]
**10.**	*Artemisia * *campestris*	*L. major*	Promastigotes	Leaves	Hydrodistillation	2.20	Essential oil	High	[17]
**11.**	*Artemisia herba-alba*	*L. major*	Promastigotes	Leaves	Hydrodistillation	1.20	Essential oil	High	[17]
**12.**	*Artemisia herba-alba*	*L. infantum.*	Amastigote	Aerial part	Methanol extract	68.25	-	Weak	[18]
		*L. major*				37.87		Moderate	
		*L. infantum*	Promastigotes			77.97		Weak	
		*L. major*				55.21		Weak	
**13.**	*Azadirachta * *indica*	*L. infantum*	Amastigotes	Leaves	Oil	15.3	Phenolics, flavonoids	Moderate	[19]
		*L. tropica*				17.6		Moderate	
**14.**	*Baphia nitida*	*L. donovani*	Promastigotes	Stem-bark	50% aqueous ethanol	34.40	Tannins, flavonoids, saponins, glycosides	Moderate	[13]
**15.**	*Bidens pilosa*	*L. donovani*	Promastigotes	Whole plant	50% aqueous ethanol	28.90	Essential oil, flavonoids, alkaloids, saponins, triterpenes	Moderate	[13]
**16.**	*Bocageopsis multiflora*	*L. amazonensis*	Promastigotes	Leaves	Ethanol	37.9	Essential oil, alkaloids	Moderate	[12]
		*L. braziliensis*				19.1		Moderate	
**17.**	*Boswellia * *serrata*	*L. donovani*	Amastigotes	Resin	Polar fractions of dichloromethane	0.88	Boswellic acids	High	[20]
**18.**	*Bridelia * *ferruginea*	*L. donovani*	Promastigotes	Leaves	50% aqueous ethanol	16.50	Flavonoids, tannins, triterpenoids	Moderate	[13]
**19.**	*Capparis * *spinosa*	*L. tropica*	Promastigotes	Fruits	Methanol	44.6	Tannins, alkaloids, saponins, terpenoids, glycosides	Moderate	[21]
					Aqueous	28.5		Moderate	
**20.**	*Cassia alata*	*L. donovani*	Promastigotes	Leaves	50% aqueous ethanol	10.10	Flavonoids, glycosides	Moderate	[22]
**21.**	*Cassia gloca*	*L. tropica*	Promastigotes	Leaves	Methanol	9.62	Flavonoids	High	[22]
**22.**	*Cassia * *sieberiana*	*L. donovani*	Promastigotes	Leaves	50% aqueous ethanol	62.90	Flavonoids, alkaloids	Weak	[23]
**23.**	*Cedrela* spp.	*L. amazonensis*	Promastigotes	Bark	Ethanol	36.8	Sesquiterpenes, triterpenes	Moderate	[22]
		*L. braziliensis*				18.2		Moderate	
**24.**	*Ceiba pentandra*	*L. donovani*	Promastigotes	Stem bark	50% aqueous ethanol	31.10	Isoflavones, sesquiterpenoids	Moderate	[13]
**25.**	*Centaurium * *erythraea*	*L. tropica*	Promastigotes	Flowering stems	n-Hexane	37.20	Phenolics, flavonoids	Moderate	[23]
		*L. major*				64.52		Weak	
**26.**	*Celtis australis*	*L. tropica*	Promastigotes	Leaves	Methanol	69.13	Flavonoids	Weak	[22]
**27.**	*Cistus crispus*	*L. major*	Promastigotes	Leaves	Methanol	84.29	Phenolics, flavonoids	Weak	[15]
		*L. infantum*			n-Hexane	82.39		Weak	
		*L. tropica*				96.82		Weak	
		*L. major*				47.29		Moderate	
**28.**	*Citrus sinensis*	*L. tropica*	Promastigotes	Leaves	Methanol	12.27	Flavonoids	Moderate	[22]
**29.**	*Cola acuminata*	*L. donovani*	Promastigotes	Stem bark	50% aqueous ethanol	47.80	Purine alkaloids, catechins, (tannins)	Moderate	[13]
**30.**	*Cola cordifolia*	*L. donovani*	Promastigotes	Stem bark	50% aqueous ethanol	25.10	Tannins, phenolics	Moderate	[13]
				Leaves		18.20		Moderate	
**31.**	*Clausena * *anisata*	*L. donovani*	Promastigotes	Roots	50% aqueous ethanol	12.10	Essential oil, indole alkaloids, coumarins	Moderate	[13]
**32.**	*Cleistopholis patens*	*L. donovani*	Promastigotes	Stem bark	50% aqueous ethanol	60.20	Flavonoids, saponins, alkaloids	Weak	[13]
**33.**	*Croton * *caudatus*	*L. donovani*	Promastigotes	Leaves	Ethyl acetate –hexane (9:1)	10.0	Terpenoids	High	[23]
			Amastigote			2.5		High	
**34.**	*Cynara * *scolymus*	*L. tropica*	Promastigotes	Stem leaf	Ethanol	80.0	-	Weak	[24]
**35.**	*Ejije bidu*	*L. amazonensis*	Promastigotes	Leaves	Ethanol	17.8	-	Moderate	[12]
		*L. braziliensis*				13.3		Moderate	
**36.**	*Erica arborea*	*L. major*	Promastigotes	Flower	Methanol	43.98	-	Moderate	[18]
		*L. infantum.*				61.27		Weak	
		*L. major*	Amastigotes			36		Moderate	
		*L. infantum.*				53.93		Weak	
**37.**	*Eryobotrya * *japonica*	*L. tropica*	Promastigotes	Leaves	Methanol	10.59	Flavonoids	Moderate	[22]
**38.**	*Eugenia * *uniflora*	*L. amazonensis*	Amastigotes	Leaves	n-Hexane	9.20	Sesquiterpenes, flavonoids	High	[25]
		*L. donovani*	Promastigotes	Seeds	50% aqueous ethanol	26.60	Essential oil, flavonoids, tannins	Moderate	[13]
**39.**	*Ferula * *communis*	*L. aethiopica*	Promastigotes	Whole parts	80% methanol	11.38	Phenolics, flavonoids	Moderate	[26]
		*L. donovani*				23.41		Moderate	
		*L. aethiopica*	Amastigotes			14.32		Moderate	
		*L. donovani*				31.12		Moderate	
**40.**	*Ficus capensis*	*L. donovani*	Promastigotes	Stem bark	50% aqueous ethanol	37.0	Alkaloids, phenolics, flavonoids	Moderate	[13]
				Leaves		88.90		Weak	
**41.**	*Glyphaea brevis*	*L. donovani*	Promastigotes	Leaves	50% aqueous ethanol	43.40	Tannins, alkaloids, flavonoids	Moderate	[13]
**42.**	*Guatteria * *Latifolia*	*L. amazonensis*	Promastigote	Branch	n-hexane fraction of ethanol	51.7	Alkaloids	Weak	[27]
**43.**	*Iresine diffusa*	*L. amazonensis*	Promastigotes	Flower	Ethanol	30.5	Sesquiterpenes, triterpenes	Moderate	[12]
		*L. braziliensis*				11.1		Moderate	
**44.**	*Jacaranda * *Glabra*	*L. amazonensis*	Promastigotes	Bark	Ethanol	29.8	-	Moderate	[12]
		*L. braziliensis*				17.4		Moderate	
**45.**	*Khaya * *grandifolia*	*L. donovani*	Promastigotes	Stem bark	50% aqueous ethanol	43.20	Alkaloids, saponins, tannins	Moderate	[13]
**46.**	*Lantana camara*	*L. amazonensis*	Amastigotes	Leaves	Dichloromethane	21.8	Terpenoids	Moderate	[28]
**47.**	*Licania * *Salicifolia*	*L. panamensis*	Amastigotes	Leaves	Ethyl acetate	9.8	Triterpenes, flavonoids	High	[29]
**48.**	*Lophira * *lanceolata*	*L. donovani*	Promastigotes	Stem bark	50% aqueous ethanol	68.60	Flavonoids, saponins, alkaloids	Weak	[13]
				Roots		66.0	Alkaloids	Weak	
**49.**	*Marrubium vulgare*	*L. infantum*	Amastigotes	Leaves	Methanol	18.64	-	Moderate	[18]
		*L. major*				32.15		Moderate	
		*L. infantum*	Promastigotes			35.63		Moderate	
		*L. major*				45.84		Moderate	
**50.**	*Mentha pulegium*	*L. infantum*	Promastigotes	Leaves	Essential oil	2.0	Menthone, pulegone	High	[30]
		*L. tropica*				2.2		High	
		*L. major*				1.30		High	
**51.**	*Mitragyna * *Inermis*	*L. donovani*	Promastigotes	Leaves	50% aqueous ethanol	21.90	Indole alkaloids, triterpenoids	Moderate	[13]
				Stem bark		28.0		Moderate	
**52.**	*Mondia whitei*	*L. donovani*	Promastigotes	Roots	50% aqueous ethanol	31.0	Glycosides	Moderate	[13]
**53.**	*Murraya * *koenigii*	*L. donovani*	Promastigotes	Stem	Petroleum ether	98.0	-	Weak	[16]
**54.**	*Oreopanax * *floribundus*	*L. panamensis*	Amastigotes	Leaves	Dichloromethane	24.6	Triterpenes	Moderate	[29]
					Ethyl acetate	23.7	Triterpenes, flavonoids	Moderate	
**55.**	*Otostegia * *integrifolia*	*L. aethiopica*	Promastigotes Amastigotes	Whole parts	80% methanol	13.03	Phenolics, flavonoids	Moderate	[31]
		*L. donovani*				17.24		Moderate	
		*L. aethiopica*				16.84		Moderate	
		*L. donovani*				14.55		Moderate	
**56.**	*Parkia * *clappertoniana*	*L. donovani*	Promastigotes	Leaves	50% aqueous ethanol	17.0	Saponins, flavonoids, Tannins	Moderate	[13]
				Stem bark		17.60	Saponins, steroids, triterpenes	Moderate	
**57.**	*Persea ferruginea*	*L. panamensis*	Amastigotes	Leaves	Ethyl acetate	25.5	Triterpenes, leucoanthocyanidins, coumarins	Moderate	[29]
**58.**	*Phoenix * *dactylifera*	*L. major*	Promastigotes	kernel	Methanol	23.0	Gallic acid	Moderate	[32]
**59.**	*Physalis * *angulata*	*L. amazonensis*	Promastigotes	Flower	Ethanol	17.6	Terpenes, phenolic acids, flavonoids	Moderate	[12]
		*L. braziliensis*				43.5		Moderate	
**60.**	*Piper * *pseudoarboreum*	*L. amazonensis*	Promastigotes	Leaves	Ethanol	31.4	Alkamides	Moderate	[33]
		*L. braziliensis*				21.3		Moderate	
		*L. guyanesis*				41.3		Moderate	
		*L. infantum*				32.3		Moderate	
**61.**	*Pistacia * *lentiscus*	*L. infantum*	Promastigotes	Leaves	Essential oil	11.28	Myrcene, α-pinene	Moderate	[23]
		*L. tropica*				23.50		Moderate	
		*L. major*				17.52		Moderate	
		*L. infantum*		Fruits	Essential oil	8.0	Limonene α-pinene	High	
		*L. tropica*				26.20		Moderate	
		*L. major*				21.42		Moderate	
**62.**	*Rosmarinus * *officinalis*	*L. infantum*	Promastigotes	Leaves	Essential oil	1.20	α-Pinene, 1,8-cineole, borneol	High	[23]
		*L. tropica*				3.50		High	
		*L. major*				2.60		High	
**63.**	*Prosopis * *juliflora*	*L. donovani*	Promastigotes	Leaves	Methanol	3.12	Saponins, tannins, flavonoids, alkaloids	High	[34]
**64.**	*Prosopis * *laevigata*	*L. amazonensis*	Amastigotes	Leaves	Aqueous	35.2	Alkaloids, anthraquinones	Moderate	[28]
**65.**	*Prunus * *armeniaca*	*L. tropica*	Promastigotes	Leaves	Ethanol	16.18	Alkaloids, phenolics, tannins, flavonoids, terpenoids, coumarins	Moderate	[35]
**66.**	*Psychotria buhitenii*	*L. panamensis*	Amastigotes	Leaves	Dichloromethane	21.5	Triterpenes, flavonoids	Moderate	[29]
					Ethyl acetate	14.1	Triterpenes, saponins, Coumarins, anthocyanins	Moderate	
					Ethanol	29.4	Saponins, phenolics, tannins, coumarins, anthocyanins	Moderate	
**67.**	*Pyrus * *communis*	*L. tropica*	Promastigotes	Leaves	Ethanol	56.68	Alkaloids, phenolics, tannins, flavonoids, terpenoids, quinones, saponins	Weak	[35]
**68.**	*Pyrus pashia*	*L. tropica*	Promastigotes	Leaves	Ethanol	60.95	Alkaloids, phenolics, tannins, flavonoids, terpenoids, quinones, saponins	Weak	[35]
**69.**	*Salvia * *clandestina*	*L. infantum*	Promastigotes	Aerial part	n-Hexane	14.11	-	Moderate	[36]
		*L. infantum*			Dichloromethane	31.57		Moderate	
		*L. tropica*				33.77		Moderate	
		*L. major*				24.56		Moderate	
**70.**	*Schinus molle*	*L. amazonensis*	Amastigotes	Leaves	Dichloromethane	25.9	Terpenoids	Moderate	[28]
					Dichloromethane: Methanol (1:1)	21.8	Terpenoids, phenolics	Moderate	
**71.**	*Schinus * *terebinthifolia*	*L. amazonensis*	Promastigotes	Fruits	n-Hexane	13.90	Triterpenes	Moderate	[29]
**72.**	*Scoparia dulcis*	*L. amazonensis*	Promastigotes	Aerial part	Ethanol	23.9	Diterpenes, triterpenes, flavonoids	Moderate	[12]
		*L. braziliensis*				25.1		Moderate	
**73.**	*Spondias mombin*	*L. donovani*	Promastigotes	Leaves	50% aqueous ethanol	81.50	-	Weak	[13]
**74.**	*Tamarindus * *indica*	*L. donovani*	Promastigotes	Leaves	50% aqueous ethanol	58.12	Phenolics, flavonoids	Weak	[13]
**75.**	*Terminalia ivorensis*	*L. donovani*	Promastigotes	Leaves	50% aqueous ethanol	24.90	Terminolic acid, quercetin, β-glycyrrhetinic acid	Moderate	[13]
**76.**	*Tessaria * *integrifolia*	*L. amazonensis*	Promastigotes	Leaves	Ethanol	54.20	Sesquiterpenes, flavonoids	Weak	[12]
		*L. braziliensis*				31.60		Moderate	
**77.**	*Thalia * *geniculata*	*L. amazonensis*	Promastigotes	Roots	Ethanol	29.8	Phytosterols	Moderate	[12]
		*L. braziliensis*				17.4		Moderate	
**78.**	*Thonningia sanguinea*	*L. donovani*	Promastigotes	Whole plant	50% aqueous ethanol	18.60	Alkaloids, tannins, flavonoids	Moderate	[13]
**79.**	*Treculia * *africana*	*L. donovani*	Promastigotes	Stem bark	50% aqueous ethanol	44.80	Catechin, cyanidin glycosides	Moderate	[13]
**80.**	*Vitex fosteri*	*L. donovani*	Promastigotes	Leaves	50% aqueous ethanol	72.40	Essential oil, flavonoids	Weak	[13]
				Stem bark		49.80		Moderate	
**81.**	*Ximenia * *americana*	*L. donovani*	Promastigotes	Stem and twigs	50% aqueous ethanol	36.10	Tannins, flavonoids, alkaloids	Moderate	[13]
**82.**	*Zanthoxylum zanthoxyloides*	*L. donovani*	Promastigotes	Roots	50% aqueous ethanol	13.50	Alkaloids, tannins, flavonoids, essential oil	Moderate	[13]
				Stem bark		45.20		Moderate	
**83.**	*Ziziphus * *spina-christi*	*L. major*	Amastigotes	Leaves	Methanol	54.6	Tannins, flavonoids, Glycosides, alkaloids, terpenoids	Moderate	[37]

**Table 4:** Anti-*Leishmania* activity of medicinal plants in vivo.

No.	Plant Species	* Leishmania * Species	Route, Dose, and Scheme of Treatment	Efficacy	Bioactive Compounds	Reference
**1.**	*Cinnamomum cassia*	Visceral leishmaniasis (*L. donovani*)	Oral: 100 mg/kg/d for 10 days	Reduction of hepatic parasitism by 80.9% and splenic parasitism by 82.9%	Cinnamaldehyde and its derivatives	[38]
**2.**	*Croton caudatus*	Visceral leishmaniasis (*L. donovani*)	Oral: 5 mg/kg/d five consecutive days	Reduction of hepatic parasitism by 65% and splenic parasitism by 69.1%	Terpenoids	[23]
**3.**	*Handroanthus serratifolius*	Cutaneous leishmaniasis (*L. amazonensis)*	Oral: 25 mg/kg/d for 10 days	24.5-fold reduction of parasite number	Lapachol	[39]
		Visceral leishmaniasis *(L. infantum)*		Reduction parasite number in spleen (4.6-fold) and liver (5.3-fold)		
**4.**	*Loranthus * *europaeus*	Cutaneous leishmaniasis (unspecific)	Topical: ointment (40%) once daily at bedtime for 6 h under occlusion for maximal 6 weeks	79.0% cure rate without side effects	Flavonoids, alkaloids, glycosides, triterpenes, phenolic acids	[40]
**5.**	*Pentalinon * *andrieuxii*	Visceral leishmaniasis (*L. donovani*)	2.5 mg/kg i.v.	Reduction of 64, 83, and 57% of parasites in the liver, spleen, and bone marrow.	Pentalinonsterol	[41]
**6.**	*Piper * *pseudoarboreum*	Cutaneous leishmaniasis (*L. amazonensis)*	Intralesional: 25 mg/kg/d for 4 days	Reduction of skin lesions by 40% and visceralization by 55%.	(E)-Piplartine	[33]
**7.**	*Prosopis * *juliflora*	Visceral leishmaniasis (*L. donovani*)	Oral: 100 mg/kg/d for 21 days	85.1% reduction of parasite number in spleen	Saponins, tannins, flavonoids, alkaloids	[34]
**8.**	*Solanum havanense*	Cutaneous leishmaniasis (*L. amazonensis)*	Intralesional: 30 mg/kg every 4 days, 5 doses	93.6% reduction of parasite number	Steroidal alkaloids, saponins, phenolics, triterpenes, coumarins	[42]
**9.**	*Solanum * *lycocarpum*	Cutaneous leishmaniasis (*L. mexicana)*	Topical: 10 μg/d for 6 weeks	71.4% reduction of parasite number	Alkaloids (solamargine, solasonine)	[43]
**10.**	*Solanum * *myriacanthum*	Cutaneous leishmaniasis (*L. amazonensis).*	Intralesional: 30 mg/kg every 4 days, 5 doses	56.8% reduction of parasite number	Steroidal alkaloids, saponins, phenolics, triterpenes, coumarins	[42]
**11.**	*Solanum * *nudum*	Cutaneous leishmaniasis (*L. amazonensis)*	Intralesional: 30 mg/kg every 4 days, 5 doses	80% reduction of parasite number	Steroidal alkaloids, saponins, phenolics, triterpenes, coumarins	[42]
**12.**	*Solanum seaforthianum*	Cutaneous leishmaniasis (*L. amazonensis)*	Intralesional: 30 mg/kg every 4 days, 5 doses	49.9% reduction of parasites in treated animals	Steroidal alkaloids, saponins, phenolics, triterpenes, coumarins	[42]
**13.**	*Tabernaemontana divaricata*	Visceral leishmaniasis (*L. donovani*)	Intraperitoneal: 5 mg/kg twice a week for 3 weeks	Decreased the hepatic parasitism by ≈30 times and splenic parasitism by ≈15 times	Voacamine	[35]
**14.**	*Urtica dioica*	Cutaneous leishmaniasis (*L. major)*	Intramuscular and intralesional: 250 mg/kg for 10 weeks	Intralesional treatment reduced lesions more than amphotericin B (control)	-	[44]
**15.**	*Ziziphus spina-christi*	Cutaneous leishmaniasis (*L. major)*	Topical: 100 and 200 mg/kg/d for 4 weeks	Reduction of lesion size by 6.4- and 8.6-fold	Tannins, flavonoids, glycosides, alkaloids, terpenoids	[37]

**Table 5:** Cytotoxic activity and selectivity index of medicinal plants in the present study (*p* = promastigote; *a* = amastigote).

No.	Plant Species	*Leishmania * Species	Part Used	Bioactive Extract/ Compounds	Cytotoxicity (CC_50_ µg/mL)	Selectivity Index (CC_50_/IC_50_)	Reference
**1.**	*Abuta grandifolia*	*L. amazonensis ^p^*	Leaves	Ethanol	15.2	0.4	[12]
		*L. braziliensis ^p^*			15.6	0.5	
**2.**	*Acanthospermum hispidum*	*L. donovani ^p^*	Whole plant	50% aqueous ethanol	55.5	1.73	[13]
**3.**	*Afzelia africana*	*L. donovani ^p^*	Stem bark	50% aqueous ethanol	232.8	3.02	[13]
**4.**	*Aniba riparia*	*L. amazonensis ^a^*	Fruits	50% aqueous ethanol	50.6	38.9	[14]
**5.**	*Annona * *senegalensis*	*L. donovani ^p^*	Leaves	50% aqueous ethanol	273.5	25.32	[13]
			Stem bark		127.9	4.60	
**6.**	*Anthocleista * *nobilis*	*L. donovani ^p^*	Leaves	50% aqueous ethanol	245.7	5.92	[13]
			Root		716.5	9.07	
**7.**	*Argemone * *mexicana*	*L. donovani ^p^*	Aerial part	Petroleum ether	52.1	0.95	[16]
**8.**	*Artemisia * *absinthium*	*L. major ^p^*	Leaves	Essential oils	11.22	7.5	[17]
**9.**	*Artemisia * *campestris*	*L. major ^p^*	Leaves	Essential oils	21.12	9.6	[17]
**10.**	*Artemisia * *herba-alba*	*L. major ^p^*	Leaves	Essential oils	11.24	9.4	[17]
**11.**	*Artemisia * *herba-alba*	*L. major ^p^*	Aerial part	Methanol	131.5	2.38	[18]
		*L. infantum ^p^*			131.5	1.86	
**12.**	*Azadirachta * *indica*	*L. infantum ^a^*	Leaves	Oil	703.8	46	[19]
		*L. tropica ^a^*			721.6	41	
**13.**	*Baphia nitida*	*L. donovani ^p^*	Stem bark	50% aqueous ethanol	990.7	28.8	[13]
**14.**	*Bidens pilosa*	*L. donovani ^p^*	Whole plant	50% aqueous ethanol	192.8	6.67	[13]
**15.**	*Bridelia * *ferruginea*	*L. donovani ^p^*	Leaves	50% aqueous ethanol	392.9	23.81	[13]
**16.**	*Bocageopsis * *multifolia*	*L. amazonensis ^p^*	Leaves	Ethanol	26.5	0.7	[12]
		*L. braziliensis ^p^*			26.7	1.4	
**17.**	*Boswellia serrata*	*L. donovani ^a^*	Resin	Polar fractions of dichloromethane	33	38	[20]
**18.**	*Capparis spinosa*	*L. tropica ^p^*	Fruits	Methanol	44.6	9.1	[21]
				Aqueous	28.5	8.4	
**19.**	*Cassia gloca*	*L. tropica ^p^*	Leaves	Methanol	1030	-	[22]
**20.**	*Cassia alata*	*L. donovani ^p^*	Leaves	50% aqueous ethanol	371.5	36.78	[13]
**21.**	*Cassia sieberiana*	*L. donovani ^p^*	Leaves	50% aqueous ethanol	62.90	0.77	[13]
**22.**	*Cedrela spp.*	*L. amazonensis ^p^*	Bark	Ethanol	66.3	1.8	[12]
		*L. braziliensis ^p^*			67.4	3.7	
**23.**	*Ceiba pentandra*	*L. donovani ^P^*	Stem bark	50% aqueous ethanol	160.7	3.32	[13]
**24.**	*Celtis australis*	*L. tropica ^p^*	Leaves	Methanol	1209	-	[22]
**25.**	*Cinnamomum cassia*	*L. donovani ^a^*	Barks	Dichloromethane fraction	No cytotoxicity at 500 µg/mL	-	[38]
**26.**	*Citrus sinensis*	*L. tropica ^p^*	Leaves	Methanol	1755	-	[22]
**27.**	*Clausena anisata*	*L. donovani ^p^*	Roots	50% aqueous ethanol	29.2	24.23	[13]
**28.**	*Cleistopholis * *patens*	*L. donovani ^p^*	Stem bark	50% aqueous ethanol	214.9	3.57	[13]
**29.**	*Cola cordifolia*	*L. donovani ^p^*	Stem bark	50% aqueous ethanol	465.6	18.55	[13]
			Leaves		465.6	25.58	
**30.**	*Cola acuminata*	*L. donovani ^p^*	Stem bark	50% aqueous ethanol	156.8	3.28	[13]
**31.**	*Cynara scolymus*	*L. tropica ^p^*	Stem leaves	Ethanol	40.0	4.96	[24]
**32.**	*Ejije bidu*	*L. amazonensis ^p^*	Leaves	Ethanol	133.5	7.5	[12]
		*L. braziliensis ^p^*			133.0	10	
**33.**	*Erica arborea*	*L. major ^p^*	Flower	Methanol	89.6	2.04	[18]
		*L. infantum ^p^*			89.6	1.46	
**34.**	*Eryobotrya * *japonica*	*L. tropica ^p^*	Leaves	Methanol	1903	-	[22]
**35.**	*Eugenia uniflora*	*L. amazonensis ^a^*	Leaves	n-Hexane	50.5	3.6	[25]
**36.**	*Eugenia uniflora*	*L. donovani ^p^*	Seed	50% aqueous ethanol	94.4	3.55	[13]
**37.**	*Ferula communis*	*L. aethiopica ^a,^*	Aerial part	80% methanol	175.22	-	[26]
		*L. donovani ^a,^*					
**38.**	*Ficus capensis*	*L. donovani ^p^*	Stem bark	50% aqueous ethanol	56.6	1.53	[13]
			Leaves		257.8	2.90	
**39.**	*Glyphaea brevis*	*L. donovani ^p^*	Leaves	50% aqueous ethanol	962.2	22.17	[13]
**40.**	*Handroanthus serratifolius*	*L. amazonensis ^p^*	Lapachol	Lapachol	3405.8	42.6	[39]
		*L. infantum ^p^*				33.0	
**41.**	*Iresine diffusa*	*L. amazonensis ^p^*	Flower	Ethanol	39.7	1.3	[12]
		*L. braziliensis ^p^*			11.1	1.7	
**42.**	*Jacaranda glabra*	*L. amazonensis ^p^*	Bark	Ethanol	18.9	6.4	[12]
		*L. braziliensis ^p^*			191.4	11	
**43.**	*Khaya * *grandifolia*	*L. donovani ^p^*	Stem bark	50% aqueous ethanol	50.1	1.16	[13]
**44.**	*Lantana camara*	*L. amazonensis ^a^*	Leaves	Aqueous	125.9	>9	[28]
**45.**	*Licania salicifolia*	*L. panamensis ^a^*	Leaves	Ethyl acetate	>200	>20.4	[29]
**46.**	*Lophira * *lanceolata*	*L. donovani ^p^*	Stem bark	50% aqueous ethanol	45.962	0.67	[13]
			Roots		38.9	0.59	
**47.**	*Marrubium * *vulgare*	*L. major ^p^*	Leaves	Methanol	107.4	2.34	[18]
		*L. infantum ^p^*			107.2	3.01	
**48.**	*Mitragyna * *inermis*	*L. donovani ^p^*	Leaves	50% aqueous ethanol	193.2	8.82	[13]
			Stem bark		424.5	15.16	
**49.**	*Mondia whitei*	*L. donovani ^p^*	Roots	50% aqueous ethanol	434.5	13.97	[13]
**50.**	*Murraya koenigii*	*L. donovani ^p^*	Stem	Petroleum ether	73.9	1.32	[16]
**51.**	*Oreopanax * *floribundus*	*L. panamensis ^a^*	Leaves	Dichloromethane	47.4	2.0	[29]
				Ethyl acetate	54.1	2.2	
**52.**	*Otostegia * *integrifolia*	*L. aethiopica ^a,^*	Aerial part	80% methanol	144.55	-	[26]
		*L. donovani ^a,p^*					
**53.**	*Parkia * *clappertoniana*	*L. donovani ^p^*	Leaves	50% aqueous ethanol	112.7	6.63	[13]
			Stem bark		42.4	2.41	
**54.**	*Persea ferruginea*	*L. panamensis ^a^*	Leaves	Ethyl acetate	>200	>7.8	[29]
**55.**	*Physalis * *angulata*	*L. amazonensis ^p^*	Flower	Ethanol	19.4	1.1	[12]
		*L. braziliensis ^p^*			17.4	0.4	
**56.**	*Piper * *pseudoarboreum*	*L. amazonensis ^p^*	Leaves	Ethanol	55.0	1.8	[33]
		*L. braziliensis ^p^*				2.6	
		*L. guyanesis ^p^*				1.3	
		*L. infantum ^p^*				1.7	
**57.**	*Prosopis juliflora*	*L. donovani ^p^*	Leaves	Methanol	0.85	0.26	[34]
**58.**	*Prosopis * *laevigata*	*L. amazonensis ^a^*	Leaves	Dichloromethane	57.0	7	[28]
**59.**	*Prunus * *armeniaca*	*L. tropica ^p^*	Leaves	Ethanol	1912.31	-	[44]
**60.**	*Psychotria * *buhitenii*	*L. panamensis ^a^*	Leaves	Dichloromethane	76.8	3.57	[29]
				Ethyl acetate	109.5	7.75	
				Ethanol	>200	>6.81	
**61.**	*Pyrus communis*	*L. tropica ^p^*	Leaves	Ethanol	1411.30	-	[35]
**62.**	*Pyrus pashia*	*L. tropica ^p^*	Leaves	Ethanol	1230.66	-	[35]
**63.**	*Schinus molle*	*L. amazonensis ^a^*	Leaves	Dichloromethane	69.7	5	[28]
				Dichloromethane: Methanol (1:1)	186.8	6	
**64.**	*Schinus * *terebinthifolia*	*L. amazonensis ^p^*	Fruits	n-Hexane	52.0	3.7	[25]
**65.**	*Scoparia dulcis*	*L. amazonensis ^p^*	Aerial part	Ethanol	71.7	3.0	[12]
		*L. braziliensis ^p^*			72.8	2.9	
**66.**	*Solanum * *lycocarpum*	*L. mexicana ^a^*	Fruits	Solamargine	1515.5	43.3	[43]
				Solasonine	1397.9	38.3	
**67.**	*Spondias mombin*	*L. donovani ^p^*	Leaves	50% aqueous ethanol	55.42	0.68	[13]
**68.**	*Tamarindus indica*	*L. donovani ^p^*	Leaves	50% aqueous ethanol	77.9	1.34	[13]
**69.**	*Terminalia ivorensis*	*L. donovani ^p^*	Leaves	50% aqueous ethanol	939.2	37.72	[13]
**70.**	*Tessaria integrifolia*	*L. amazonensis ^p^*	Leaves	Ethanol	119.2	2.2	[12]
		*L. braziliensis ^p^*			120.0	3.8	
**71.**	*Thalia geniculata*	*L. amazonensis ^p^*	Roots	Ethanol	50.7	1.7	[12]
		*L. braziliensis ^p^*			50.4	2.9	
**72.**	*Thonningia sanguinea*	*L. donovani ^p^*	Whole plant	50% aqueous ethanol	286.1	15.38	[13]
**73.**	*Treculia africana*	*L. donovani ^p^*	Stem bark	50% aqueous ethanol	172.0	3.84	[13]
**74.**	*Urtica dioica*	*L. major ^p^*	Leaves	Aqueous	4500	4.4	[44]
**75.**	*Vitex fosteri*	*L. donovani ^p^*	Leaves	50% aqueous ethanol	114.4	1.58	[13]
			Stem bark		420.3	8.44	
**76.**	*Ximenia * *americana*	*L. donovani ^p^*	Stem and twigs	50% aqueous ethanol	42.3	1.17	[13]
**77.**	*Zanthoxylum zanthoxyloides*	*L. donovani ^p^*	Roots	50% aqueous ethanol	247.1	18.30	[13]
			Stem bark		583.5	12.91	
**78.**	*Ziziphus * *spina-christi*	*L. major ^a^*	Leaves	Methanol	563.3	10.31	[37]

**Table 6:** Anti-*Leishmania* activity of isolated natural compounds.

No.	Compound Name	*Leishmania * Species	Stage	Assay	Values (IC50)	Data Analysis (Activity)	Authors
**1**	2,3-Dihydrobenzofuran	*L. amazonensis*	Promastigotes	In vitro	1.04 µg/mL	High	[45]
			Amastigotes		1.4 µg/mL	High	
**2**	Dehydrodieuginol	*L. amazonensis*	Promastigotes	In vitro	42.4 µg/mL	Moderate	[31]
**3**	Erytro-manassatin A	*L. amazonensis*	Promastigotes	In vitro	35.4 µg/mL	Moderate	[46]
			Amastigotes		20.4 µg/mL	Moderate	
**4**	Threo-manassatin A	*L. amazonensis*	Promastigotes	In vitro	17.6 µg/mL	Moderate	[46]
			Amastigotes		16.0 µg/mL	Moderate	
**5**	Epipinoresinol-4-O-β-D-glucopyranoside	*L. major*	Promastigotes	In vitro	36.5 µg/mL	Moderate	[47]
**6**	Calanolide E1	*L. major*	Promastigotes	In vitro	36.5 µg/mL	Moderate	[48]
**7**	Calanolide E2	*L. major*	Promastigotes	In vitro	29.1 µg/mL	Moderate	[48]
**8**	Caffeic acid	*L. infantum*	Promastigotes	In vitro	12.5 µg/mL	Moderate	[49]
			Amastigotes		21.9 µg/mL	Moderate	[50]
**10**	Capsaicin	*L. infantum*	Promastigotes	In vitro	5.01 µg/mL	High	[51]
			Amastigotes		24.2 µg/mL	Moderate	
**11**	Cassine	*L. amazonensis*	Promastigotes	In vitro	25.2 µg/mL	Moderate	[52]
**12**	Spectaline	*L. amazonensis*	Promastigotes	In vitro	15.8 µg/mL	Moderate	[52]
**13**	Berberine	*L. donovani*	Promastigotes	In vitro	4.8 µg/mL	High	[53]
**14**	Colchicoside	*L. major*	Promastigotes	In vitro	0.2 µg/mL	High	[54]
			Amastigotes		4.0 µg/mL	High	
**15**	Bisabolol	*L. donovani*	Visceral leishmaniasis	In vivo	39.4 µM	Moderate	[55]
**16**	2-Demethyl colchicine	*L. major*	Promastigotes	In vitro	0.5 µg/mL	High	[54]
			Amastigotes		10.2 µg/mL	Moderate	
**17**	3-Demethyl colchicine	*L. major*	Promastigotes	In vitro	0.4 µg/mL	High	[54]
			Amastigotes		11.1 µg/mL	Moderate	
**18**	Cornigerine	*L. major*	Promastigotes	In vitro	0.8 µg/mL	High	[54]
			Amastigotes		11.9 µg/mL	Moderate	
**19**	Piperine	*L. infantum*	Promastigotes	In vitro	3.03 µg/mL	High	[51]
**20**	Colchicine	*L. major*	Promastigotes	In vitro	0.4 µg/mL	High	[54]
			Amastigotes		8.7 µg/mL	High	
**21**	N-deacetyl-N-formyl colchicine	*L. major*	Promastigotes	In vitro	0.5 µg/mL	High	[54]
			Amastigotes		10.2 µg/mL	Moderate	
**22**	Colchifoline	*L. major*	Promastigotes	In vitro	0.7 µg/mL	High	[54]
			Amastigotes		14.0 µg/mL	Moderate	
**23**	Demecolcine	*L. major*	Promastigotes	In vitro	0.7 µg/mL	High	[54]
			Amastigotes		14.8 µg/mL	Moderate	
**24**	Staurosporine	*L. amazonensis*	Promastigotes	In vitro	0.08 µM	High	[56]
			Amastigotes		10.0 µM	High	
		*L. donovani*	Promastigotes		2.1 µM	High	
**25**	7-Oxostaurosporine	*L. amazonensis*	Promastigotes	In vitro	3.6 µM	High	[56]
			Amastigotes		0.1 µM	High	
		*L. donovani*	Promastigotes		0.6 µM	High	
**26**	4′-Demethylamine-4′- oxostaurosporine	*L. amazonensis*	Promastigotes	In vitro	17.1 µM	Moderate	[56]
			Amastigotes		2.0 µM	High	
**27**	Streptocarbazole B	*L. amazonensis*	Promastigotes	In vitro	10.4 µg/mL	Moderate	[56]
			Amastigotes		2.5 µg/mL	High	
**28**	3-O-acetylspectaline	*L. donovani*	Promastigotes	In vitro	25.9 µg/mL	Moderate	[53]
**29**	3-O-acetylcassine	*L. donovani*	Promastigotes	In vitro	30.3 µg/mL	Moderate	[53]
**30**	Soranjidiol	*L. amazonensis*	Promastigotes	In vitro	16.3 J/cm^2^	Moderate	[57]
**31**	Epigallocatechin 3-*O*- gallate	*L. infantum*	Visceral leishmaniasis	In vivo	ED50 = 12.4 mg/kg/day	Moderate	[58]
**32**	5-Chlorosoranjidiol	*L. amazonensis*	Promastigotes	In vitro	13.8 J/cm^2^	Moderate	[58]
**33**	Bisoranjidiol	*L. amazonensis*	Promastigotes	In vitro	15.2 J/cm^2^	Moderate	[58]
**34**	Gallic acid	*L. major*	Promastigotes	In vitro	23.0 µg/mL	Moderate	[32]
**35**	Calanolides E1	*L. infantum.*	Amastigotes	In vitro	37.1 µM	Moderate	[48]
**36**	Calanolides E2				29.1 µM	Moderate	
**37**	Apigenin	*L. amazonensis*	Promastigotes	In vitro	23.7 µM	Moderate	[59]
			Amastigotes		4.3 µM	High	
**38**	2′-hydroxyflavanone	*L. amazonensis*	Promastigotes	In vitro	20.5 µM	Moderate	[60]
			Amastigotes		3.09 µM	High	
**39**	5,7,3′,4′-tetrahydroxy- 6,8-diprenylisoflavone	*L. amazonensis*	Promastigotes	In vitro	2.7 µM	High	[61]
			Amastigotes		1.1 µM	High	
**40**	Brachydin B	*L. braziliensis*	Promastigotes	In vitro	7.05 µM	High	[62]
**41**	Brachydin C	*L. amazonensis*	Promastigotes	In vitro	10.0 µM	High	[62]
			Amastigotes		6.25 µM	High	
		*L. braziliensis*	Promastigotes		8.8 µM	High	
**42**	Ursolic acid	*L. amazonensis*	Promastigotes	In vitro	6.2 µg/mL	High	[63]
		*L. donovani*	Amastigotes		1.8 µM	High	
**43**	Aplysulphurin	*L. donovani*	Amastigotes	In vitro	3.1 µM	High	[64]
**44**	Tetrahydroaplysulphurin-1	*L. donovani*	Amastigotes	In vitro	3.5 µM	High	[64]
**45**	Membranolide	*L. donovani*	Amastigotes	In vitro	9.7 µM	High	[64]
**46**	Apigenin	Cutaneous leishmaniasis	Cutaneous leishmaniasis	In vivo	ED50 = 0.73 mg/kg	High	[65]
**47**	Darwinolide	*L. donovani*	Amastigotes	In vitro	11.2 µM	Moderate	[63]
**48**	Pukalide aldehyde	*L. donovani*	Amastigotes	In vitro	1.9 µM	High	[66]
**49**	Epigallocatechin 3-*O*- gallate	*L. infantum*	Amastigotes	In vitro	2.6 µM	High	[58]

## Data Availability

Not applicable.

## References

[1] World Health Organization (2020). Leishmaniasis Fact-Sheets.

[2] Center for Food Security and Public Health (2009). Leishmaniasis (Cutaneous and Visceral).

[3] Bereket A., Mihiretu A. (2017). Leishmaniasis: A review on parasite, vector and reservoir host. Health Sci. J..

[4] Roberts L., Janovy J., Schmidt G. (2009). Foundations of Parasitology.

[5] Gagandeep K., Bhawana R. (2014). Comparative analysis of the omics technologies used to study antimonial, amphotericin B, and pentamidine resistance in *Leishmania*. J. Parasitol. Res..

[6] Ghorbani M., Farhoudi R. (2018). Leishmaniasis in humans: Drug or vaccine therapy?. Drug Des. Develop. Ther..

[7] Jawed J., Majumdar S. (2018). Recent trends in *Leishmania* research: A therapeutic perspective. J. Infect. Epidemiol..

[8] Haldar A., Sen P., Roy S. (2011). Use of antimony in the treatment of leishmaniasis: Current status and future directions. Mol. Biol. Int..

[9] Murugan N., Natarajan D. (2018). Bionanomedicine for antimicrobial therapy—A case study from *Glycosmis pentaphylla* plant-mediated silver nanoparticles for control of multidrug-resistant bacteria. Lett. Appl. NanoBioSci..

[10] Et-Touys A., Bouyahyal A., Fellah H., Mniouil M., El Bouryl H., Dakka N., Bakri Y. (2017). Antileishmanial activity of medicinal plants from Africa: A review. Asian Pac. J. Trop. Dis..

[11] Santos D., Coutinho C., Madeira M., Bottino C., Vieira R., Nascimento S., Rodrigues C. (2008). Leishmaniasis treatment a challenge that remains: A review. Parasitol. Res..

[12] Arévalo-Lopéz D., Nelida N., Ticona J.C., Limachi I., Salamanca E., Udaeta E., Paredes C., Espinoza B., Serato A., Garnica D. (2018). Leishmanicidal and cytotoxic activity from plants used in Tacana traditional medicine (Bolivia). J. Ethnopharmacol..

[13] Ohashi M., Amoa-Bosompem M., Kwofie K., Agyapong J., Adegle R., Sakyiamah M., Ayertey F., Owusu K., Tuffour I., Atcholog P. (2018). In vitro antiprotozoan activity and mechanisms of action of selected Ghanaian medicinal plants against *Trypanosoma*, *Leishmania*, and *Plasmodium* parasites. Phytother. Res..

[14] Costa L., Alves M., Brito L., Abi-Chacra E., Barbosa-Filho J., Gutierrez S., Barreto H., Carvalho F. (2021). In vitro antileishmanial and immunomodulatory activities of the synthetic analogue riparin E. Chem. Biol. Interact..

[15] Bouyahya A., Et-Touys A., Dakka N., Fellah H., Abrini J., Bakri Y. (2018). Antileishmanial potential of medicinal plant extracts from the North-West of Morocco. Beni-Suef Univ. J. Basic Appl. Sci..

[16] Sultana S. (2021). In vitro antileishmanial activity of three medicinal plants: *Argemone mexicana* Murraya Koenig and *Cinnamomum tamala* against miltefosine resistant promastigotes of *Leishmania donovani* parasites. Int. J. Pharm. Pharmaceut. Sci..

[17] Mathlouthi A., Belkessan M., Sdiri M., Fethi-Diouani M., Souli A., El-Bok S., Ben-Attia M. (2018). Chemical composition and anti-leishmania major activity of essential oils from *Artemesia* spp. grown in central Tunisia. J. Essent. Oil Bear. Plants.

[18] Eddaikra N., Boudjelal A., Sbabdji M.A., Eddaikra A., Boudrissa A., Bouhenna M.M., Smain C., Zoubir H. (2019). Leishmanicidal and cytotoxic activity of Algerian medicinal plants on *Leishmania major* and *Leishmania infantum*. J. Med. Microbiol. Infect. Dis..

[19] Cesa S., Sisto F., Zengin G., Scaccabarozzi D., Kokolakis A., Scaltrito M., Grande R., Locatelli M., Cacciagrano F., Angiolella L. (2019). Phytochemical analyses and pharmacological screening of neem oil. S. Afr. J. Bot..

[20] Greve H., Kaiser M., Mäser P., Schmidt T. (2021). Boswellic acids show in vitro activity against *Leishmania donovani*. Molecules.

[21] Mohammad R., Sareh J., Katrin E., Massumeh N., Maryam S., Mehrdad K., Sam K. (2020). Cytotoxic and antileishmanial effect of various extracts of *Cappris spinose* L. Turk. J. Pharm. Sci..

[22] Shah N.A., Khan M.R., Nigussie D. (2016). Phytochemical, antioxidant and anti-*Leishmania* activity of selected Pakistani plants. J. Pharmacol. Clin. Res..

[23] Bouyahya A., Bakri Y., Belmehdi O., Et-Touys A., Abrini J., Dakka N. (2019). Phenolic extracts of *Centaurium erythraea* with novel antiradical, antibacterial and antileishmanial activities. Asian Pac. J. Trop. Dis..

[24] Ahmet Y., Tulay A., Sahra C., Husniye K., Eda T., Cuneyt B. (2021). Assessment of in vitro activity of *Cynara scolymus* extracts against *leishmania tropica*. Kafkas Univ. Vet. Fak. Derg..

[25] Beatriz M., Adriana B., Sonia A., Eliana R., Eric U., João H., Márcia D., Susan P., Luiz F. (2019). Ethnopharmacology study of plants from Atlantic forest with leishmanicidal activity. Evid. Based Complement. Alternat. Med..

[26] Nigatu H., Belay A., Ayalew H., Abebe B., Tadesse A., Tewabe Y., Degu A. (2021). In vitro antileishmanial activity of some Ethiopian medicinal plants. J. Exp. Pharmacol..

[27] Ferreira C., Passos C., Soares D., Costa K., Rezende M., Lobao A., Pinto A., Hamerski L., Saraiva E. (2017). Leishmanicidal activity of alkaloids-rich fraction from *Guatteria latifolia*. Exp. Parasitol..

[28] Ronna D., Lianet M., Abel P., Heike V., Fausto R., Cesar I., Alejandra R. (2017). In vitro antileishmanial activity of Mexican medicinal plants. Heliyon.

[29] Wilson C., Sara R., Fernando A., Andres F., Cristian H., Ivan D., Juan C., Isabel V. (2020). Antileishmanial and cytotoxic activities of four Andean plant extracts from Colombia. Vet. World.

[30] Bouyahya A., Et-Touys A., Bakri Y., Talbaui A., Fellah H., Abrini J., Dakka N. (2017). Chemical composition of *Mentha pulegium* and *Rosmarinus officinalis* essential oils and their antileishmanial, antibacterial and antioxidant activities. Microb. Pathog..

[31] Rodrigues L., Barbosa-Filho J., de Oliveira M., do Nascimento N., Borges F., Mioso R. (2016). Synthesis and antileishmanial activity of natural dehydrodieugenol and its mono- and dimethyl ethers. Chem. Biodivers..

[32] Albakhit S., Khademvatan S., Doudi M., Forutan-Rad M. (2016). Antileishmanial activity of date (*Phoenix dactylifera* L.) fruit pit extract in vitro. Evid. Based Complement. Altern. Med..

[33] Ticona J., Bilbao-Ramos P., Flores N., Dea-Ayuela M., Bolás-Fernández F., Jiménez I., Bazzocchi I. (2020). (E)-Piplartine isolated from *Piper pseudoarboreum*, a lead compound against leishmaniasis. Foods.

[34] Mutile M., Muli M., Muita G. (2021). Safety and efficacy of *Prosopis juliflora* leaf extract as a potential treatment against visceral leishmaniasis. Iran. J. Parasitol..

[35] Nargis S., Naveeda A., Attiya I., Asma A., Huma F. (2020). Evaluation of safety, antileishmanial and chemistry of ethanolic leaves extracts of seven medicinal plants: An in-vitro study. Open Chem. J..

[36] Et-Touys A., Fellah H., Sebti F., Mniouil M., Elboury H., Talbaoui A., Bakri Y. (2016). In vitro antileishmanial activity of extracts from endemic Moroccan medicinal plant *Salvia verbenaca* (L.) Briq. ssp *verbenaca* Maire (*S. clandestina* Batt. non L.). Eur. J. Med. Plants.

[37] Albalawi A. (2021). Antileishmanial activity of *Ziziphus spina-christi* leaves extract and its possible cellular mechanisms. Microorganism.

[38] Afrin F., Chouhan G., Islamuddin M., Want M., Ozbak H., Hemeg H. (2019). *Cinnamomum cassia* exhibits antileishmanial activity against *Leishmania donovani* infection in vitro and in vivo. PLoS Negl. Trop. Dis..

[39] Araújo I., de Paula R., Alves C., Faria K., Oliveira M., Mendes G., Dias E., Ribeiro R., Oliveira A., Silva S. (2019). Efficacy of lapachol on treatment of cutaneous and visceral leishmaniasis. Exp. Parasitol..

[40] Khalifa E., Adil A., Banaz M., Zinah A., Wasnaa S. (2017). Topical 40% *Loranthus europaeus* ointment as an alternative medicine in the treatment of acute cutaneous leishmaniasis versus topical 25% podophyllin solution. J. Cosmetics Dermatol. Sci. Appl..

[41] Gupta G., Peine K., Abdelhamid D., Snider H., Shelton A., Rao L., Kotha S., Huntsman A., Varikuti S., Oghumu S. (2015). A novel sterol isolated from a plant used by Mayan traditional healers is effective in treatment of visceral leishmaniasis caused by *Leishmania donovani*. ACS Infect. Dis..

[42] Paul C., Janssens J., Abel P., Osmany C., Arianna Y., Alexis D., Wagner V., Lianet M. (2018). Efficacy of four *Solanum* spp. extracts in an animal model of cutaneous leishmaniasis. Medicines.

[43] Lezama-Dávila M., McChesney D., Bastos K., Miranda A., Tiossi F., da Costa J., Bentley D., Gaitan-Puch M., Marquez I. (2016). A new antileishmanial preparation of combined solamargine and solasonine heals cutaneous leishmaniasis through different immunochemical pathways. Antimicrob. Agents Chemother..

[44] Badirzadeh A., Heidari-Kharaji M., Fallah-Omrani V., Dabiri H., Araghi A., Salimi Chirani A. (2020). Antileishmanial activity of *Urtica dioica* extract against zoonotic cutaneous leishmaniasis. PLoS Negl. Trop. Dis..

[45] De Castro O., Brito L., de Moraes A., Amorim L., Sobrinho-Júnior E., de Carvalho C., Rodrigues K., Arcanjo D., Cito A., Carvalh F. (2017). In vitro effects of the neolignan 2,3-dihydrobenzofuran against *Leishmania amazonensis*. Basic Clin. Pharmacol. Toxicol..

[46] Brito J., Passero L., Bezerra-Souza A., Laurenti M., Romoff P., Barbosa H., Ferreira E., Lago J. (2019). Antileishmanial activity and ultrastructural changes of related tetrahydrofuran dineolignans isolated from *Saururus cernuus* L. (*Saururaceae*). J. Pharm. Pharmacol..

[47] Maia M., Silva J., Nunes T., Sousa J., Rodrigues G., Monteiro A., Tavares J., Rodriqes K., Junior F., Scotti L. (2020). Virtual screening and the in vitro assessment of the antileishmanial activity of lignans. Molecules.

[48] Silva L., Gomes K., Costa-Silva T., Romanelli M., Tempone A., Sartorelli P., Lago J. (2020). Calanolides E1 and E2, two related coumarins from *Calophyllum brasiliense* Cambess. (*Clusiaceae*), displayed in vitro activity against amastigote forms of *Trypanosoma cruzi* and *Leishmania infantum*. Nat. Prod. Res..

[49] Bortoleti B., Tomiotto-Pellissiera F., Gonçalves M., Miranda-Sapla M., Assolini J., Carloto A., Lima D.M., Silveira G.F., Almeida R.S., Costa I.N. (2019). Caffeic acid has antipromastigote activity by apoptosis-like process; and anti-amastigote by TNF-α/ROS/NO production and decreased of iron availability. Phytomedicine.

[50] Garcia A., Oliveira D., Amaral A., Jesus J., Rennó Sodero A., Souza A., Supuran C., Vermelho A., Rodrigues I., Pinheiro A. (2019). *Leishmania infantum* arginase: Biochemical characterization and inhibition by naturally occurring phenolic substances. J. Enzyme Inhib. Med. Chem..

[51] Vieira-Araújo F., Macedo Rondon F., Pinto Vieira Í., Pereira Mendes F., Carneiro de Freitas J., Maia de Morais S. (2018). Synergism between alkaloids piperine and capsaicin with meglumine antimoniate against *Leishmania infantum*. Exp. Parasitol..

[52] Lacerda R., Freitas T., Martins M., Teixeira T., da Silva C., Candido P., Oliveira R., Junior C., Bolzani V., Danuello A. (2018). Isolation, leishmanicidal evaluation and molecular docking simulations of piperidine alkaloids from *Senna spectabilis*. Bioorg. Med. Chem..

[53] De Sarkar S., Sarkar D., Sarkar A., Dighal A., Staniek K., Gille L., Chatterjee M. (2018). Berberine chloride mediates its antileishmanial activity by inhibiting *Leishmania* mitochondria. Parasitol. Res..

[54] Azadbakht M., Davoodi A., Hosseinimehr S., Keighobadi M., Fakhar M., Valadan R., Farindnia R., Emami S., Azadbakht M., Bakhtiyari A. (2020). Tropolone alkaloids from *Colchicum kurdicum* (Bornm.) Stef. (Colchicaceae) as the potent novel antileishmanial compounds, purification, structure elucidation, antileishmanial activities, and molecular docking studies. Exp. Parasitol..

[55] Corpas-López V., Merino-Espinosa G., Díaz-Sáez V., Morillas-Márquez F., Navarro-Moll M., Martín-Sánchez J. (2016). The sesquiterpene (–)-α-bisabolol is active against the causative agents of Old World cutaneous leishmaniasis through the induction of mitochondrial-dependent apoptosis. Apoptosis.

[56] Cartuche L., Sifaoui I., López-Arencibia A., Bethencourt-Estrella C., San Nicolás-Hernández D., Lorenzo-Morales J., Pinero J., Marrero A., Fernandez J. (2020). Antikinetoplastid activity of indolocarbazoles from *Streptomyces sanyensis*. Biomolecules.

[57] Dimmer J., Cabral F., Sabino C., Silva C., Núñez-Montoya S., Cabrera J., Ribeiro M. (2019). Natural anthraquinones as novel photosensitizers for antiparasitic photodynamic inactivation. Phytomedicine.

[58] Inacio J., Fonseca M., Almeida-Amaral E. (2019). (−) -Epigallocatechin 3-O-gallate as a new approach for the treatment of visceral leishmaniasis. J. Nat. Prod..

[59] Sen R., Chatterijee M. (2011). Plant-derived therapeutics for the treatment of leishmaniasis. Phytomedicine.

[60] Gervazoni L., Gonçalves-Ozório G., Almeida-Amaral E. (2018). 2′-Hydroxyflavanone activity in vitro and in vivo against wild-type and antimony-resistant *Leishmania amazonensis*. PLoS Negl. Trop. Dis..

[61] Pereira I., Mendona D., Tavares G., Lage D., Ramos F., Oliveirada-Silva J., Antinarelli L., Machado A., Caravalh A., Salustiano I. (2020). Parasitological and immunological evaluation of a novel chemotherapeutic agent against visceral leishmaniasis. Parasite Immunol..

[62] Rocha V., Quintino C., Ferreira Queiroz E., Marcourt L., Vilegas W., Grimaldi G., Furrer P., Allemann E., Wolfender J., Soares M. (2018). Antileishmanial activity of dimeric flavonoids isolated from *Arrabidaea brachypoda*. Molecules.

[63] Das S., Ghosh S., De A., Bera T. (2017). Oral delivery of ursolic acid-loaded nanostructured lipid carrier coated with chitosan oligosaccharides: Development, characterization, in vitro and in vivo assessment for the therapy of leishmaniasis. Int. J. Biol. Macromol..

[64] Shilling A., Witowski C., Maschek J., Azhari A., Vesely B., Kyle D., Amsler C., McClintock J., Baker B. (2020). Spongian diterpenoids derived from the antarctic sponge *Dendrilla antarctica* are potent inhibitors of the leishmania parasite. J. Nat. Prod..

[65] Fonseca-Silva F., Canto-Cavalheiro M., Menna-Barreto R., Almeida-Amaral E. (2015). Effect of apigenin on *Leishmania amazonensis* is associated with reactive oxygen species production followed by mitochondrial dysfunction. J. Nat. Prod..

[66] Thomas S., von Salm J., Clark S., Ferlit S., Nemani P., Azhari A., Rice C., Wilson N., Kyle D., Baker B. (2018). Keikipukalides, furanocembrane diterpenes from the antarctic deep sea octocoral Plumarella *delicatissima*. J. Nat. Prod..

[67] Colares A., Almeida-Souza F., Taniwaki N., Souza S., da Costa J., Calabrese K., Abreu-Silva A. (2013). In vitro antileishmanial activity of essential oil of *Vanillosmopsis arborea* (*Asteraceae*) Baker. Evid. Based Complement. Alternat. Med..

[68] Gervazoni L., Barcellos G., Ferreira-Paes T., Almeida-Amaral E. (2020). Use of natural products in leishmaniasis chemotherapy: An overview. Front. Chem..

[69] Machado M., Dinis A., Santos-Rosa M., Alves V., Salgueiro L., Cavaleiro C., Sousa M. (2014). Activity of *Thymus capitellatus* volatile extract, 1, 8-cineole and borneol against *Leishmania* species. Vet. Parasitol..

[70] Yamamoto E., Campos B., Jesus J., Laurenti M., Ribeiro S., Kallas E., Fernandes M., Gomes G., Silva M., Seessa D. (2015). The effect of ursolic acid on *Leishmania (Leishmania) amazonensis* is related to programed cell death and present therapeutic potential in experimental cutaneous leishmaniasis. PLoS ONE.

[71] Sen R., Bandyopadhyay S., Dutta A., Mandal G., Ganguly S., Saha P., Chatterjee M. (2007). Artemisinin triggers induction of cell-cycle arrest and apoptosis in *Leishmania donovani* promastigotes. J. Med. Microbiol..

[72] Sen R., Ganguly S., Saha P., Chatterjee M. (2010). Efficacy of artemisinin in experimental visceral leishmaniasis. Int. J. Antimicrob. Agents.

[73] Hajaji S., Sifaoui I., Arencibia A., Batlle M., Jimenez I., Bazzocchi I., Valladares B., Akkari H., Morales J., Pinero J. (2018). Leishmanicidal activity of α-bisabolol from Tunisian chamomile essential oil. Parasitol. Res..

[74] Brenzan M., Santos A., Nakamura C., Filho B., Ueda Nakamura T., Young M., Correa A., Junior J., Diaz J., Cortez D. (2012). Effects of (−) mammea A/BB isolated from *Calophyllum brasiliense* leaves and derivatives on mitochondrial membrane of *Leishmania amazonensis*. Phytomedicine.

[75] Guyatt G., Sackett D., Cook D. (1994). Users’ guides to the medical literature II: How to use an article about therapy or prevention. J. Am. Med. Assoc..

[76] Dey S., Mukherjee D., Chakraborty S., Mallick S., Dutta A., Ghosh J., Swapana N., Maiti S., Ghorai N., Singh C.B. (2015). Protective effect of *Croton caudatus* Geisel leaf extract against experimental visceral leishmaniasis induces proinflammatory cytokines in vitro and in vivo. Exp. Parasitol..

